# Supplementary material for: Are carbon emissions trading and green financial instruments synergistic? -Comprehensive quantitative research based on content analysis
Source: PLoS One. 2024 Mar 7;19(3):e0298601. doi: 10.1371/journal.pone.0298601 (PMC10919601; doi:10.1371/journal.pone.0298601)
Supplement: S1 Data — (ZIP) [file pone.0298601.s001.zip › Data/地级市Z_F工作报告.zip!/╔╜╬≈/┬└┴║╩╨/└┤╫╘╣1⁄2╓┌║┼í╛┬φ┐╦╩2╛▌═°í┐_2014.docx]

现在，我代表市人民政府向大会作工作报告，请予审议，并请市政协委员和其他列席人员提出意见。

2013年工作回顾

过去一年，在市委的坚强领导下，我们深入贯彻落实党的十八大和十八届三中全会精神，按照“打基础、利长远、惠民生”的总体要求，着力推动转型跨越发展，经济社会各项工作取得了显著成绩。

——积极应对复杂困难局面，调结构、促转型，市域经济保持平稳增长。2013年，全市地区生产总值完成1228.6亿元，增长9.5%；公共财政预算收入完成163.98亿元，增长15.5%；规模以上工业企业增加值完成875.6亿元，增长12%；固定资产投资完成872.9亿元，增长26.4%；社会消费品零售总额完成344亿元，增长14.2%；外贸进出口总额完成7.48亿美元，增长30.1%；居民消费价格涨幅3.0%，控制在年度目标3.5%以内；城镇登记失业率2.1%，低于4.2%的控制目标。地区生产总值、公共财政等4项主要经济指标增幅位居全省前列。

——以综改试验为统领、以项目建设为抓手，经济结构调整迈出重要步伐。扎实推进综改试验区建设，编制完成转型综改2013—2015年三年实施方案和年度行动计划，“五规合一”、户籍管理、行政审批、中小微企业融资、矿产资源产权交易等五项重大改革顺利推进，扶贫开发、资源转化、生态建设、城镇化等10个方面重大事项全面启动，20个重大项目进展顺利。全面加快项目建设，全年共铺开重点工程173个，完成投资1445亿元，在省重点工程“六位一体”12项考核指标中，我市获得5个第一、2个第二、1个第三，走在全省前列，有力促进了结构调整和产业升级。传统产业继续做大做强，临县霍煤1000万吨煤矿、兴县肖家洼1000万吨煤矿、兴县中铝100万吨氧化铝、孝义信发180万吨氧化铝、汾阳杏花村酒业集中发展区、交城国锦2×30万千瓦电厂等项目基本建成或正式投产。孝义信发铝系二期、汾阳国峰2×30万千瓦电厂等项目全面开工建设。中阳中澳生态铝循环经济一体化等项目落地奠基。高新技术产业取得重大突破，吕梁军民融合协同创新研究院在应用微小卫星、无人系统、能源互联网及新材料工业等领域取得较大进展，特别是“天河二号”落户吕梁，为我市成为全省、全国重要的运算中心奠定了基础。全年新兴产业增加值增速高出工业增加值7个百分点，占到工业增加值的20.6%，创历史新高。第三产业健康发展，孝义义乌商品交易国际博览城基本建成，沃尔玛、红星美凯龙、居然之家等现代物流企业进驻吕梁，商贸流通现代化水平进一步提高。全市第三产业增加值达到296.5亿元，占 GDP 比重较上年增长1.9个百分点。

——农业基础地位不断巩固，农业农村面貌发生明显变化。粮食总产量达11.6亿公斤，实现“九连增”。“三农”支出达到113.96亿元，同比增长12.38%。“8+2”农业产业振兴工程顺利起步，建成核桃和红枣标准化管理示范园区3.5万亩、绿色谷子生产基地2.3万亩，新发展林下中药材1.3万亩、设施蔬菜2.52万亩，建设一级种薯和绿色商品薯基地各1万亩，发展窑洞食用菌6015孔、574.5万棒。扶贫攻坚扎实开展，启动“百企千村”产业扶贫工程，50家省市企业与10个贫困县、1000个贫困村结对开发，组建开发公司20家，实施各类项目63个。易地扶贫搬迁、片区开发、整村推进等扶贫开发项目顺利推进，实现12.7万低收入贫困人口稳定脱贫。造林绿化深入推进，全年共造林77.4万亩，栽植核桃林50.9万亩。在汾阳市成功举办了第七届世界核桃大会。

——基础设施建设日趋完善，发展后劲进一步增强。城镇化建设稳步推进。《吕梁城市总体规划》（2013—2030）得到省政府批复，吕梁新区主干路网、河道整治以及供水、供气、供热管网建设全面铺开，回迁安置房及部分配套设施相继开工。实施大县城建设项目211项，完成投资44.75亿元。保障性安居工程开工30335套，建成24648套。全市城区集中供热普及率达到67.91%，燃气普及率达到76%，污水处理率达到75.6%，生活垃圾处理率达到51.3%。城镇化率达到43.11%，较上年提高1.5个百分点。基础设施不断完善。吕梁机场正式通航，中南、太兴及吕临支线铁路加快推进，西纵、环城两条高速基本具备通车条件。完成农村公路建设338公里。中部引黄工程进展顺利，龙门供水取水枢纽主体工程完工，千年水库主要工程建设任务基本完成，坪底供水工程大坝主体全面封顶。新打机井200眼，增加灌溉面积5万亩。煤层气勘探开发利用迈出重大步伐，地面抽采量达到15亿立方米，输气管网架构基本形成。

——社会事业同步推进，城乡统筹取得新进展。义务教育全面发展，交城、文水、方山、岚县完成薄弱学校改造任务，石楼、兴县、临县在城镇学校扩容改造和农村寄宿制学校附属生活设施建设方面取得实质性进展。全年新改扩建城区幼儿园21所、农村幼儿园75所，高中阶段毛入学率达到93.5%。高考二本B类以上达线人数首次突破万人大关。科技创新成果斐然，申请专利963件，全社会研究与试验发展经费达到4亿元，增长18%。山西吕梁国家农业科技园区正式获批，建成1个国家级企业技术中心、2个省级工程技术研究中心。医药改革继续深化，全市13个县级综合医院全部达到二级甲等标准，圆满完成县级公立医院改革试点任务。新农合参合人数达到276.3万人，参合率达到98.46%，筹资标准提高到每人340元。新农保参保人数达到170.9万人，领取待遇人数达到36万人。文化事业不断繁荣，10个省级非遗项目通过评审，晋剧《刘胡兰》获全国舞台艺术最高奖“文华剧目奖”。吕梁慈善总会正式成立。卫生扶贫、物价管理、防震减灾等工作受到国家表彰或中央领导肯定，为吕梁赢得了荣誉。

——城乡居民收入快速增长，人民生活质量得到新改善。落实增收措施。上调最低工资标准，发布企业工资指导线，落实带薪休假制度；培育富民产业，加大补贴力度，积极培训和转移劳动力，鼓励以工补农、以城带乡，推进机关定点扶贫、干部包村下乡。通过一系列有效措施，城乡居民收入较快增长，其中城镇居民人均可支配收入达20145元，增长9.9%；农民人均纯收入达6067元，增长13.1%。完善社会保障。城乡居民基础养老金每人每月增加10元；企业退休人员基本养老金提高10%；城镇居民医保财政补助标准提高40元；城乡低保补助标准每人每月分别提高30元、24元；失业人员失业保险金标准提高15%以上；财政筹资570万元，将市属48户国有特困企业的3043名职工纳入医疗保险和大病保险。改善生态环境。12个县（市）和吕梁市区平均优良天数比例达到91.8%，市区 PM2.5 日均浓度达标率为78.9%，全省排名第二。

——安全生产持续向好，和谐发展取得新成绩。坚持把安全生产作为最大的民生工程和必须坚守的底线，坚决落实政府监管和企业主体责任，深入开展安全生产大检查和隐患排查治理，保持打非治违高压态势，安全生产形势持续明显好转，事故起数和死亡人数同比分别下降2.62%、18.85%。深入开展“平安吕梁”建设，大力实施“六六创安”工程，积极推进“一社区三中心”建设，积极排查化解社会矛盾。扎实开展食品安全专项检查，深入推进“六五”普法。进一步健全社会治安防控体系，有效处置各类突发事件，社会保持和谐稳定。

——切实转变工作作风，政府自身建设取得新突破。坚决执行“八项规定”，有效遏制“四风”，全年共查处违反“八项规定”案件231件，处理267人。党政机关停止新建楼堂馆所，全面清理规范办公用房。搭建公共资源交易监督平台，交易总成本比分散交易降低15%以上。深入推进“两集中、两到位”，全市行政审批项目由448项减少为132项。自觉执行人大及其常委会的决议、决定，支持人民政协履行职能，全年共办理人大代表建议156件、政协委员提案466件。

各位代表，以上成绩来之不易。这是省委、省政府坚强领导、亲切关怀的结果，是市委统揽全局、科学决策的结果，是市人大、市政协有力监督、大力支持的结果，是全市广大干部群众同心同德、团结奋斗的结果。在此，我代表市人民政府，向全市人民、解放军指战员、武警官兵、公安干警和驻市单位，向各民主党派、人民团体，向尽心履职的各位人大代表和政协委员，向所有关心支持吕梁改革发展的各级领导和各界朋友，表示崇高的敬意和衷心的感谢！

在总结成绩的同时，我们也清醒认识到工作中还存在很多问题和不足：转型综改尚未取得重大突破，体制机制有待进一步创新；“一煤独大”产业格局远未改变，经济发展方式粗放，创新水平低下，抵御市场风险的能力严重不足；社会事业发展滞后，城乡一体化进程缓慢，城乡居民收入在全省处于垫底位置；环境保护和节能减排形势严峻，生态文明建设任重道远；安全生产基础仍不牢固，安全责任和保障措施有待进一步强化。同时，在政府职能转变、作风建设、廉洁勤政等方面，仍然存在不容忽视的问题，在个别领域和部门还很突出。对此，我们将高度重视，不回避、不推诿，采取得力措施，认真加以解决。

2014年工作安排

2014年是全面贯彻落实党的十八大和十八届三中全会精神的重要一年，是实施“十二五”规划的关键一年。

今年政府工作的指导思想是：认真贯彻党的十八届三中全会精神，全面落实市委三届五次全会暨全市经济工作会议决策部署，牢牢坚持“打基础、利长远、惠民生”总体要求，以转型综改试验区建设为统领，以经济结构调整为主线，以扶贫攻坚为要务，以民生改善为根本，攻坚克难，逆势奋进，着力推动经济平稳健康发展，保持社会和谐稳定，为开创各项工作新局面，为走出资源型地区转型跨越发展新路、全面建成小康社会努力奋斗。

2014年我市经济社会发展主要预期指标是：地区生产总值增长9％，全社会固定资产投资增长23％，社会消费品零售总额增长14.5％，公共财政预算收入增长8％，城镇居民人均可支配收入增长12%，农民人均纯收入增长15％，城镇新增就业岗位4.1万个，城镇登记失业率控制在4.2%以内，居民消费价格涨幅控制在3.5%左右。

约束性指标是：万元地区生产总值综合能耗下降3.6%、二氧化碳排放量下降3.7%；二氧化硫、化学需氧量、氨氮、氮氧化物和烟尘、工业粉尘排放量完成省下达任务，万元工业增加值用水量降幅3.2％。

各位代表，确立以上目标，既是完成省委、省政府考核任务的基本要求，更是我市经济社会平稳健康发展的内在需要，我们一定要坚定信心，埋头苦干，奋力进取，主动作为，确保目标任务圆满实现。当前，宏观经济形势严峻复杂，我市传统支柱产业受到巨大冲击，区域金融环境异常趋紧，企业生产经营困难很大，有的经济指标出现多年未有的负增长。面对严峻局势，我们要保持定力，增强耐力，激发动力，焕发活力。保持定力，就是要坚定看到丰富的资源禀赋依然是我市经济发展的最大优势，我市经济积极向好的基本面没有改变，工业经济整体迈进“大矿时代”，基础设施有了根本性改善，云计算、新能源、新材料等新兴产业开始崛起，具备了抗御风险的经济体量和物质条件。现在的困难是较高发展水平上的暂时曲折，是多年高速增长之后的合理回落，没有理由因为市场的正常波动而惊慌失措，甚至丧失信心。增强耐力，就是要客观地看到随着全国经济进入结构调整阵痛期和增长速度换挡期，能源原材料价格理性回归，经济增长速度适当放缓，是符合经济规律的必然现象，我们要立足于这样的预判分析形势、定位工作、谋划发展，从而减少好大喜功的盲目，消除急于求成的浮躁，用更加理性、客观、从容的态度，扎扎实实做好工作。激发动力，就是要清醒地看到市场经济的严酷性、经济结构的脆弱性和加快转型的紧迫性，利用市场的倒逼机制，真正强化转型意识，坚定转型目标，加快转型步伐，化危为机，着力打造吕梁经济升级版。焕发活力，就是要敏锐地把握国家和省委、省政府全面深化改革、推进转型综改、刺激经济增长带来的巨大发展机遇，不失时机地搭上内陆开放、中部崛起特别是新型城镇化的快车，培育新亮点，积蓄新优势，实现新跨越。我们坚信，在省委、省政府和市委的坚强领导下，只要全市上下切实增强忧患意识和进取意识，凝心聚力、提振信心，精诚团结、众志成城，以上项目、保增长，抓转型、惠民生，重安全、促稳定为重要抓手，就一定能够促进经济社会平稳健康发展，推动各项工作再上新台阶。

今年，在全面做好各项工作的同时，重点要抓好以下十个方面的工作：

一、促进经济平稳健康增长

保持市域经济平稳健康发展是今年工作的重中之重。面对困难，我们要以更加饱满的热情、更加昂扬的斗志、更加切实有效的措施，聚精会神谋发展，群策群力度难关。

千方百计营造宽松发展环境。认真落实省政府“煤炭17条”、“保障工业运行12条”和支持中小微企业发展的“财政15条”、“金融12条”，以及市政府出台的“煤炭10条”、“扶持中小微企业20条”等一系列政策措施，全力为企业减负卸压、增强活力。充分发挥市场调控作用，抓好现有企业提产增效。依法依规加快复工复产验收，帮助企业尽快恢复生产经营。制定出台更为积极的新兴产业、高新技术产业优惠政策，支持引进原创发明专利和技术，着力培育新的经济增长点。实行领导一对一挂牌帮扶机制，帮助企业强化内部管理，降低生产成本，提高产品的市场竞争力。鼓励企业转变经营理念，创新促销方式，增强市场意识。加强行业组织和协调，帮助企业稳定客户，开拓市场，特别是煤焦行业要加快建立与大客户的稳固联系，实现长期合同供货，切实增强抗御市场波动的能力。强化发改、经信、统计等经济综合部门对经济运行分析工作，创新服务方式，切实发挥好对经济运行的分析、研判、监测和预警作用。

坚定不移抓好项目建设。以深入开展“项目见效年”活动为契机，项目建设“六位一体”全面推进。今年要完成项目储备20000亿元，项目签约1500亿元，项目落地1500亿元，项目开工1500亿元，项目建设1450亿元，项目投产1500亿元，全年固定资产投资突破1000亿元，力争达到1100亿元，在量的增长和质的提升上都有一个新的跨越。要不断深化领导包联项目制度，完善项目考核奖惩办法，坚持重点工程月调度机制，积极开展项目集中审批月活动，实行项目先期预审协调和并联审批机制，以及“一站式服务”、“一个窗口办理”机制和严肃问责制，高效、规范推进项目建设。要进一步加大招商引资力度。按照国家产业政策和环保政策，依托自身优势、针对自身不足，瞄准优势产业、潜力产业、短板产业，以商招商、以企招商、以园招商，力求在新兴产业、重点领域取得新突破。要加快实施人才强市战略，大力引进一批高端人才和紧缺人才。要深化区域合作，积极发展飞地经济，主动对接京津产业转型。

全力以赴激发企业活力。落实梯度扶持政策，实施“星火”创业工程，新创办1500户中小微企业；实施“小升规”企业壮大工程，新增加30户营业收入2000万元以上规模企业；实施“小巨人”培育工程，新增15户营业收入亿元以上“小巨人”企业。同时，积极支持企业进行技术改造和创新挖潜，增强企业内生活力。

毫不动摇推进园区建设。坚持把园区作为集聚生产要素、优化产业布局、发展循环经济的重要载体，采取有力措施，逐步解决目前我市各类园区“乱、散、弱、小”的问题，打造企业集聚发展的坚实平台。要对现有各类园区进行清理整顿，整合要素，提升水平，形成特色，创立品牌，按“一县一区”的原则申报省级经济开发区。要重点推进孝义市国家级循环经济城市创建工作，加快兴县中铝循环经济园区率先发展。

多措并举创新金融服务。积极构建政府引导、市场主导的政企银合作平台，深入推进对金融机构的考核评比，努力实现银企共赢。支持新的金融机构落户吕梁，鼓励现有金融机构扩容增量，延伸服务覆盖面。鼓励引导民间资本进入金融服务领域，大力发展村镇银行、小额贷款公司、融资性担保公司，积极创造条件，开办地方银行。鼓励金融机构创新服务，加快推出适合企业发展的各类金融产品，着力缓解企业融资困难。支持企业通过资产、订单、专利抵押和商标专用权质押等形式，拓宽融资渠道。加快企业上市步伐，支持企业发行债券，扩大私募基金规模，提高直接融资能力。大力整顿金融秩序，依法打击非法集资，积极防范和化解区域性金融风险，努力营造良好的金融生态环境。

二、加快转变经济发展方式

转变经济发展方式既是长期的战略方针，又是当前的紧迫任务，要立足现实基础，着眼未来发展，着力打造“四基三带一核心”，逐步形成科技创新引领，新兴产业、传统产业两翼齐飞的雁阵式经济发展新格局，走出一条资源型经济低碳发展、循环发展、绿色发展、可持续发展新路子。

做大做强传统产业，着力打造“四大产业基地”，夯实经济平稳健康发展基础。

一要推进煤电一体。在加快建设现代化矿井的同时，全力争取低热值煤发电项目，规划发展一批坑口电厂，实现“输煤”向“输电”转变、单一矿井向煤电一体转型。确保交城国锦2×30万千瓦电厂并网发电；加快推进汾阳国峰2×30万千瓦、文水国金1×35万千瓦电厂建设；开工建设临县霍州煤电2×35万千瓦、孝义晋能新阳2×35万千瓦、兴县华电锦兴2×35万千瓦、中阳大唐桃园2×35万千瓦电厂；积极争取离石大土河热电联产、兴县晋能电厂、交口华瑞电厂项目取得“路条”。编制完成全市火电发展规划，力争到“十二五”末，全市电力装机容量达到1000万千瓦，初步扭转我市煤多电少、单极发展的被动局面，着力将我市打造成煤电一体化发展产业基地。

二要坚持焦化并举。巩固焦炭企业兼并重组成果，全市焦炭总量控制在4500万吨以下，单企规模达到200万吨级，园区（集中区）焦炭生产占到全市总产能的85%以上。大力发展煤化工产业，不断延伸煤化工产业链，实现焦油、粗苯、焦炉煤气等焦炭副产物集中加工和综合利用，并逐步实现由初级产品向碳材料和精细化工转变。重点抓好交城宏特超高功率石墨电极、碳纤维、粗蒽深加工，孝义金州针状焦配套焦油深加工，孝义晋茂粗苯加氢精制等项目，着力将我市打造成焦化一体化发展产业基地。

三要延伸冶金加工。在发展冶金下游产业上做文章，进一步推进交城、岚县、交口等生铁铸造加工基地发展壮大，同时重点加强铝系产业发展，依托交口、孝义、兴县三个铝镁集中加工区，着力延伸铝土矿—氧化铝—电解铝—铝型材—高端铝加工产业链条。抓紧推进交口道尔200万吨铝系产业园、兴华科技70万吨铝基加工、信发肥美延伸铝材加工，孝义信发二期180万吨氧化铝、兴县华兴二期100万吨氧化铝、柳林森泽煤矸石提取100万吨氧化铝、中阳中澳生态铝循环经济一体化等项目建设，重点规划上马一批附加值高、市场前景好的铝材加工项目，着力将我市打造成生态铝循环经济一体化发展产业基地。

四要实现白酒集聚。推动我市白酒生产由产量扩张向市场扩张转变、分散发展向集中发展转变、散白酒向品牌白酒转变。加快完善杏花村酒业集中发展区建设，支持文水散白酒集中发展，支持白酒行业成立战略联盟，鼓励中低端白酒企业与汾酒集团合作，改造提升生产工艺，创新管理手段，稳步扩展消费市场，着力将我市打造成白酒行业规模、品牌一体化发展产业基地。

培育壮大新兴产业，着力打造“三大产业集聚带”，构建市域经济新的增长极。

一要提升装备制造业水平。确保实现孝义与上海合作的新能源客车一期项目整车下线，同时重点抓好孝义金晖矿山煤机设备，汾阳联通玻璃钢管道、东晟刹车盘，交城国正高铁动车制动盘、正泰特种机电装备，文水华一重工列车车轮车轴、光华高效离心球墨铸管等重点项目建设，着力打造装备制造产业集聚带。

二要培育新能源、新材料产业。加快推进大唐岚县河口和王狮风电、云顶山文水和离石风电、文水晋能太阳能电池及太阳能组件、孝义太子光伏电站、汾阳中节能光伏发电等新能源项目，积极扶持孝义宏天源铝系列材料、任氏华普瑞锂离子电池材料，方山安华汇丰节能环保材料、离石凌云龙盟石头造纸、交城国利天能反射镜、汾阳昊阳太阳能备板膜等新材料项目建设，着力打造新能源、新材料产业集聚带。

三要推动旅游产业成长。全面启动全市旅游产业发展规划编制工作，积极推进旅游资源整合，支持条件成熟的胜溪湖森林公园、柏洼山等景区申报国家4A级旅游景区。依托关帝山国家森林公园和庞泉沟国家自然保护区，整合庞泉沟、北武当山、南阳沟、云顶山、苍儿会、千年等自然生态景区，发展太原都市圈绿色生态休闲度假板块。以兴县黑峪口、临县碛口镇和曲峪镇、柳林三交镇、石楼东辛关和黄河石楼湾为节点南北延伸，形成“四点一线”的黄河民俗风情板块。链接则天故里、汾酒工业园、卦山、玄中寺等旅游景点，开发与晋中晋商文化、太原都市文化、临汾根祖文化差异互补的特色文化板块。突出红军东征、晋绥革命、吕梁英雄谱三大主题，形成以兴县、临县、石楼、交城和文水为主的红色经典板块，着力打造红色、古色、绿色旅游产业集聚带。

同时，高度重视服务业发展，加快吕梁天源物流中心、汾阳阳城商贸物流区、孝义丰邦农商贸批发市场、红星美凯龙等服务业重点项目建设，积极推进15分钟便民商圈建设，培育新的消费市场和消费习惯，把更多的消费留在吕梁，确保服务业增加值增幅高于全市经济增长速度。

加快推进创新驱动，着力打造“高新科技核心区”，增强转型跨越发展核心竞争力。

引深与国防科技大学合作，全力支持吕梁军民融合协同创新研究院发展，积极推进无人机产业基地、柳林李家湾光电子产业园、岚县江川国威新材料等“一院三基地”建设，着力实现高性能云计算、应用微小卫星、无人机系统和能源互联网的产业化，同时积极做好全省煤炭产业清洁安全低碳高效发展科技攻关项目试点工作和低碳创新行动，争取更多的高科技攻关项目落户我市，着力打造以离石区为中心、辐射带动全市各县（市、区）的高新科技核心区。

各位代表，发展是第一要务，作为欠发达地区，经济建设这个中心任何情况下都不能动摇和偏离。越是困难的时候，我们越要发扬艰苦奋斗的吕梁精神，凝神聚力，万众一心，推动吕梁经济破浪前行！

三、深入推进转型综改区建设

改革是今年政府工作的主旋律，转型综改区建设是我们全面深化改革的总抓手，要按照省委、省政府“转型攻坚年”的部署，解放思想，敢闯敢试，突破体制障碍，化解发展难题，向改革要动力、靠转型促发展。

按照转型综改2014年行动计划，整体推进重大改革、重大事项、重大项目，力争在重点领域和关键环节取得重大突破。建立完善循环经济发展促进机制。进一步创新循环经济发展模式，着力构建企业小循环、园区中循环、区域大循环三级联动的循环经济发展机制。大力支持孝义市创建国家级循环经济示范县（市）。构建促进就业长效机制。继续推进政府购买基层公益性岗位工作，实施就业培训、就业援助和就业信息服务“三个全覆盖”，建立统一的人力资源市场。探索建立生态环境保护修复多元投入和补偿机制。加快发展环保产业,鼓励和引导社会资本参与生态环境治理，构建环境保护多元投入机制。统筹使用矿山生态恢复治理保证金投向污染河流综合整治。深化教育、医药卫生体制改革。整合教育资源，改革办学体制，大力推进义务教育均衡发展。深入推进公立医院综合改革，推动基本药物制度向非政府办基层医疗机构延伸试点。健全科技创新体制机制。坚持产学研协同创新，围绕产业链部署创新链，按照创新链配置资源链，强化自主创新能力建设，加快实现由要素驱动向创新驱动转变。创新农业生产经营机制。全力推进“8+2”农业产业化振兴计划，加快家庭经营、集体经营、合作经营、公司经营等共同发展的新型农业经营体系建设。深化财税管理体制改革。实行全口径预算管理，实施全面规范的预决算公开，自觉接受社会监督。合理划分市、县事权和支出责任，进一步提高民生支出比例。

全面启动20个重大事项、30个重大项目建设，支持和推进孝义、柳林综改试点和扩权强县试点工作，先行先试，率先发展，充分发挥示范引领作用，带动全市县域经济快速发展。

四、扎实做好“三农”工作

始终把“三农”工作作为重中之重，以增加农民收入为核心，大力培育富民产业，深入推进扶贫攻坚，确保全市农业农村经济再上一个新台阶。

发展特色现代农业。强力推进“8+2”农业产业化。市级财政安排1亿元专项资金，重点扶持建设9.5万亩核桃、红枣示范园，8万亩绿色杂粮示范片，4.5万亩绿色马铃薯基地，新发展12.5万亩林下经济，种植食用菌1400万棒，完成肉牛改良3万头、人工种草1.3万亩、引进湖羊种羊4500只，建设3000亩西兰花出口基地。同时，积极扶持一批农民专业合作社、家庭农场、专业大户。市财政注入3000万元“助保金”，启动市级“助保贷”业务，培育壮大加工龙头，推动农产品加工企业集群发展，大力推进兴县农业园区、临县城北红枣园区、汾阳核桃产业园区等项目建设。全市农产品加工总量达到130万吨，销售收入突破100亿元。加大新型职业农民培训力度，继续实施农业科技示范展示工程，扎实推进山西吕梁国家农业科技园区建设，努力提高农业综合生产能力。突出抓好粮食生产，开展粮食高产创建活动，重视粮食流通工作，确保粮食总量、结构平衡和粮食安全。

深入推进扶贫攻坚。扎实推进百企千村产业扶贫开发工程。年内再启动200个贫困村的产业扶贫开发，同时积极引导民营企业和省外企业参与产业扶贫开发，真正把产业扶贫建成富民增收工程。切实抓好千村万人就业培训素质提升工程，贫困劳动力培训就业人数达到1万人以上。依托国家“阳光工程”、退耕区劳动力技能培训和科技入户项目，完成引导性培训15万人，新转移5万人。坚持易地扶贫搬迁与城镇化建设、产业开发、旧村开发利用和完善社会保障相结合，年内完成易地扶贫搬迁1万人以上，新扶持整村推进村100个。力争片区扶贫开发项目在10个贫困县（区）全覆盖、彩票公益金扶贫项目在6个国定贫困县全覆盖。争取再有13万低收入贫困人口稳定脱贫。

改善农村人居环境。以建设家园美、田园美、生态美、生活美的美丽乡村为目标，分层次、有步骤启动实施“四大工程”，即：以农村基础设施和公共服务为重点的完善提升工程，以采煤沉陷区治理、易地搬迁、危房改造为重点的农民安居工程，以垃圾污水治理为重点的乡村清洁工程、环境整治工程，以美丽乡村建设为重点的宜居示范工程，年内率先建设一批省、市级美丽宜居示范村。今年再改造农村危房1万户，解决7.4万农村人口和1.7万名师生的饮水安全问题。同时，在确保完成省“五件实事”的基础上，集中力量抓好我市“便民六件实事”，如期兑现向全市人民的承诺。

各位代表，让农民过上好日子，是各级政府义不容辞的责任。要舍得投入强农业，倾注真情富农民，决不让农村拖全面小康的后腿，决不让贫困代代相传！

五、积极稳妥推进城镇化

新型城镇化建设是拉动经济增长的重要引擎。认真贯彻刚刚召开的全省城镇化工作会议精神，按照《吕梁市城市总体规划》，积极稳妥推进城镇化进程，力争全市城镇化率再提高1.8个百分点。

加快形成离柳中方城镇组群。以离石、柳林、中阳、方山同城化发展为目标，努力打造太原都市圈西部区域中心。要尽快实现柳林李家湾、中阳金罗、离石交口和方山峪口等区域与中心城区功能衔接和互补；统一规划建设离柳中方水电气暖、通信、学校、医院等各类公益事业，推进离柳中方一体化发展。坚定不移有序推进吕梁新区建设，加快完善水、暖、电、气等市政基础设施,抓好城市生活污水、生活垃圾处理设施和地下管网、防洪排涝等体系建设。全力推进离石、方山安置房建设，年内完成吕梁大道（一期）、新区与环城高速及机场连接线、八条街道、九座桥梁、站前广场、北川河整治（一期）等工程，加快推进离石、方山安置区幼儿园、中小学和吕梁长途汽车站等工程建设，搭起新区城市基础设施主框架。同时，坚持新区建设与中心城区提质并举，年内完成市区控制性详规编制工作，严厉打击违法违规建设行为。大力实施扩容提质工程，年内开工建设第二污水处理厂，新建自来水厂投入使用，彻底解决城区供水不足的问题；打通滨河东路、呈祥路两条断头路，提升改造凤山公园循环路，新建四座过街天桥，维修改造城区小街小巷30条，缓解城区交通压力。大力实施增绿提质工程，对原有公园、绿地提升改造，新增绿地面积15000平方米。稳步推进住房保障工程，新建公共租赁住房2144套，棚户区改造13400套。

推动孝义、汾阳率先实现城镇化。依托孝汾平介灵城镇组群布局，明确孝义、汾阳两市“引领吕梁山、融入太原圈”的发展定位，进一步做好两市与孝汾平介灵城镇组群的规划衔接。坚持组团式发展，突出区位、资源和文化优势，加快建设功能完备、产业强劲的新型城市，不断提升两市作为区域中心的辐射带动功能，引导吕梁城镇化向纵深发展，形成太原都市圈的二级核心区和吕梁城镇化的先导示范区。

加快大县城、重点镇建设。全程启动和推进各县（市、区）城镇体系规划和城镇总体规划的编制修编工作。积极开展县城基础设施、公共服务设施、公园绿地、中心街市、居住社区“五项建设”和景观风貌、环境卫生“两项整治”，提高大县城吸纳人口、优化服务、集聚产业的综合承载能力，切实解决好各县城新增人口就业、入学、就医、社保等问题，真正实现公共服务均等化，促进群众生活方式向现代化、健康化转变，努力提高人民群众的幸福指数。继续加强百镇建设，支持有条件的重点镇、中心村就地、就近城镇化，探索一条符合吕梁实际、符合百姓意愿的新型城镇化建设模式，加快城乡一体化、均衡化发展进程。

六、大力加强基础设施建设

基础设施建设滞后仍然是制约我市经济社会发展的瓶颈。加快基础设施建设既是打基础的核心，又是利长远的根基，更是惠民生的保障。

加快形成立体交通网络。今年是我市重点铁路、公路“通车年”。中南铁路、太兴铁路以及西纵高速、环城高速、太佳高速黄河大桥等工程年内要确保通车。要加快战略装车点建设，列入省重点工程的20个铁路战略装车点（含专用线），年内要全部开工建设，并争取与铁路同步投运。抓紧做好已列入全省高速规划的西纵高速南段、兴县黑峪口—静乐丰润、汾阳—石楼3条高速公路的前期工作，力争方山大武—祁县城赵高速开工建设。积极争取开通直达太原城际快速列车。加快完善吕梁机场配套设施，增加北京首都国际机场航班，形成早晚对开格局；在增开上海航线的基础上，再争取开通长沙、三亚，西安、广州航线。

加快水利基础设施建设。继续加快龙门供水、千年水库等重点水利设施建设，年内龙门供水工程输水隧洞实现全线贯通，千年水库下闸蓄水。积极推进峪口沟中型水库、离石北寒沟等5座小型水库工程的前期工作，做好中部引黄工程建设协调保障,启动受益9县（市、区）小水网规划审批后续设计工作。加大农田水利、水库（水闸）除险加固、中小河流治理等民生工程建设力度，完成农田实灌面积155.5万亩。不断完善和健全防汛抗旱应急体制和管理机制，全面提升防汛抗旱能力。

加快电网建设。完成中南铁路配套的1座500千伏、4座220千伏输变电工程和6座牵引站供电工程。完成文水国金、汾阳国峰两个220千伏电厂送出工程。完成交口桃红坡220千伏变电站和文水南安、孝义西盘粮、汾阳城中、兴县花子、柳林刘家山、交口温泉等110千伏输变电工程，进一步完善吕梁电网网架结构。大力实施农网改造升级工程，逐步消除农村“低电压”现象。

加快“气化吕梁”建设。加快煤层气由勘探向开发转变，力争两年内实现地面煤层气总产能25亿立方米，煤矿瓦斯抽采量16亿立方米。加快交口、石楼、方山、中阳天然气、煤层气民用普及水平，争取年底实现县城区“气化”全覆盖。加快天然气、煤层气综合开发利用，重点在发电和工业燃料、压缩和液化煤层气等领域的利用技术推广，逐步提高清洁能源工业化利用水平。

七、全面发展各项社会事业

加快社会事业建设步伐，坚持重投入、补短板、兜底线、保民生，不断提高人民生活水平和质量，让全市人民共享发展改革成果。

优先发展教育事业。启动第二轮学前教育三年行动计划，新建城镇标准化幼儿园21所，改造农村幼儿园44所。继续推进义务教育标准化建设和改造薄弱学校，孝义、离石通过国家义务教育均衡发展评估验收。启动特殊教育提升计划，新建临县、柳林特殊教育学校。启动基础教育信息化建设工程和教育教学提升工程，建成200所具有示范带动意义的特色学校。基本普及高中阶段教育。加快建设县级职教中心，做好吕梁职业技术学院计划招生工作，努力构建具有吕梁产业特色、与转变经济发展方式相适应的现代职业教育体系。支持高等教育健康发展。继续做好家庭困难学生的资助工作，确保每一个贫困家庭学生上得起学。

大力发展医疗卫生事业。继续深化医药卫生体制改革，以取消“以药补医”为重点，实现县级公立医院改革全覆盖，同时推动基本药物制度向基层非政府办医疗机构延伸。创新医疗服务，有效缓解医患矛盾。进一步提高基本医疗保障服务水平，将新农合人均筹资标准提高至390元，政策范围内住院费用报销比例达到70%。积极推进新农合大病医疗保险。加快中心医院、中医院、妇幼保健院等市级医疗卫生机构建设，积极筹建吕梁护理职业学院，启动建设市、县120医疗紧急救援机构。新建、改扩建一批基层医疗卫生服务机构。做好人口计生工作。深入开展爱国卫生运动，加快吕梁市创建国家级卫生城市步伐。

不断完善社会保障体系。推进社会保障城乡一体化，建立统一的居民基本养老保险制度，积极支持养老机构建设。城镇职工医保、城镇职工和居民大病保险实行市级统筹。继续提高企业退休人员基本养老金，鼓励有条件的县（市、区）提高城乡居民基础养老金等各项社会保险待遇水平。城镇居民基本医保财政补助标准提高到人均320元，城乡低保月均保障标准分别提高25元、22元，农村五保户集中供养、分散供养标准每人每年分别提高1900元、500元。推动医保付费方式改革，实现医疗保险省内异地就医即时结算。鼓励发展社会养老机构。加大对困难企业职工、下岗失业人员的救助力度，持续增加农村贫困人口的低保覆盖面，力争到“十二五”末，全市享受农村低保人数占贫困人口的比例达到50%。扶持发展各类慈善事业，形成全方位社会救助体系。稳定居民消费价格总水平，抓好价格调节基地建设，努力增加市场供应，推进平价商店建设和市场建设。

加快文化兴市步伐。进一步深化文化体制机制改革，推动政府部门由办文化向管文化转变。完善公共文化服务体系，加快市级“五馆一院”、县级“三馆一院”建设。建立政府购买公共文化服务长效机制，深入开展送戏下乡、送文化下乡活动，不断扩大公共文化产品覆盖范围。广泛开展群众性文化生活，推进文艺精品创作，加快发展文化产业，努力促进文化与科技、旅游等产业深度融合，进一步提高文化产业在国民经济中的比重。强化文化市场和广电安全监督管理，加大网络舆论引导和管控力度。加强文物和非物质文化遗产保护，依法打击破坏和盗窃文物等违法犯罪行为。

统筹发展其他事业。深入开展双拥工作，发展人民防空事业。落实民族宗教政策，做好外事、侨务、对台等工作，发展妇女儿童、老龄、残疾人和红十字会等事业，加强未成年人和其他弱势群体保护工作，抓好气象、科普、档案、史志等工作。搞好第三次经济普查和地理国情普查。加快农村乡镇体育健身广场和社区体育设施建设步伐。

八、深入推进生态文明建设

生态文明建设关系人民生活、关系未来发展。必须切实加强生态环境保护和建设，建立健全生态文明制度体系，建设美丽、宜居吕梁。

强力推进节能降耗。推进工业、建筑、交通、公共机构等领域节能降耗，做好煤炭、焦化、冶金、电力、化工、建材等重点行业节能工作。深入开展能效对标活动，推进千家企业节能低碳行动，引导企业实施节能技术改造。严格执行固定资产投资项目节能评估审查。

突出抓好治污减排。深入开展大气污染防治专项行动。加快重点行业环保设施升级改造，推动各县（市）和吕梁中心城区煤改气，扩大集中供暖范围，抓好加油站油气治理、餐饮业油烟污染治理和城区扬尘污染防治，做好黄标车淘汰和机动车环保检测治理等重点工作。全力推进水体治理，启动文峪河、三川河、磁窑河、湫水河、蔚汾河、岚河、双池河等7条主要河流沿线12县（市、区）水环境质量综合整治规划工程建设项目；推进以污水处理提质扩容、截污管网扩展为主的城市环境基础设施建设；加强柳林泉补给区等饮用水水源地保护工作。扎实开展生态环保创建活动，年内完成柳林县和汾阳市省级环保模范城市验收工作；完成省、市级生态村镇创建任务；加大农村环境集中连片整治、畜禽养殖场和屠宰场的排污整治力度。

加大造林绿化和生态治理修复力度。依托三北防护林、退耕还林、天然林保护等国家重点工程，结合全省造林绿化吕梁现场推进会筹备工作，深入开展造林绿化，大力推进吕梁山生态脆弱区植被恢复工程，实施好通道绿化、交通沿线荒山绿化、环城绿化、村庄绿化、矿区植被恢复等增绿工程。年内完成造林63万亩，新发展核桃林30万亩。完善生态补偿机制，深入开展地质灾害调查评估、综合防治和工程治理。持续加大水土资源保护，继续推进“五百千”重点水保生态治理工程，年内完成水土流失治理任务40万亩。全年取水总量控制在5.78亿立方米以内。

各位代表，有了绿水青山，就是金山银山。我们要切实转变观念，付出最大努力，为吕梁面貌的根本改变，为子孙后代的幸福生活，向污染宣战，向绿色进军！

九、全力维护社会安全稳定

安全稳定先于一切、高于一切、大于一切，是执政为民的紧要大事，是吕梁发展的根本大局，必须夯实基层基础，完善机制体制，为发展改革和人民安居乐业提供有力保障。

全力做好安全生产工作。坚持安全发展理念，牢固树立“红线”意识，敬畏生命、敬畏责任、敬畏制度。深入开展知责履责活动，严格落实“党政同责、一岗双责、齐抓共管”的安全生产责任制。加快隐患排查治理体系建设，深入开展安全生产专项整治，保持打非治违高压态势，按照“全覆盖、零容忍、严执法、重实效”要求，加大督查检查力度，及时发现和消除安全隐患。坚决杜绝重特大安全事故，遏制较大事故、减少一般事故，促进全市安全生产形势持续稳定好转。建立健全农产品质量全过程追溯管理体系，全面强化食品药品监管，切实保障人民群众“舌尖上的安全”。

全力维护社会稳定。深入开展“平安吕梁”建设，继续巩固“三大活动”成果，加强社会治安管理，完善打防控一体化的社会治安防控体系，年内实现“一村一警”全覆盖。创新群众工作方法，进一步规范和畅通群众诉求表达渠道，有效预防和化解社会矛盾。加强应急管理和防灾减灾能力建设，妥善应对、及时处置各类突发事件和群体性事件，全力维护政治稳定、社会安定。

十、努力建设人民满意的政府

完成繁重的改革建设任务，必须全面加强政府自身建设，努力构建法治政府、服务政府、责任政府、廉洁政府和学习型政府。

加快政府职能转变。进一步简政放权，继续取消和下放行政审批事项，遏制权力寻租，提高行政效率。对保留的行政审批项目，一律公布项目清单，清单以外的一律不予实施。认真做好各级调整和下放行政审批事项的承接工作，继续深化行政审批“两集中、两到位”改革，确保审批高效、企业方便、群众满意。扎实抓好事前事中事后监管，防止缺位失位,着力堵塞缝隙和漏洞。按时间节点完成市县两级政府机构改革，整合机构职能，规范机构设置，严格控制编制，提高行政效率。加快推进事业单位分类改革。

深入推进依法行政。自觉接受人大及其常委会的法律监督、工作监督，主动接受人民政协民主监督。严格执行人大及其常委会的决议决定，认真办理人大代表、政协委员的建议和提案。广泛听取民主党派、工商联、无党派人士的意见建议，充分发挥工会、共青团、妇联等人民团体作用。支持法院、检察院依法独立公正行使职权。完善科学民主决策机制，增强公共政策制定的透明度和公众参与度。依法公开政府信息，提高政府工作透明度，让权力在阳光下运行。发展基层民主，做好第十届村委会换届选举工作。深化普法教育，加强政府法制工作，建立政府法律顾问制度，落实行政执法责任制、执法案卷评查制和执法责任追究制，形成办事依法、遇事找法、解决问题用法、化解矛盾靠法的良好法治环境。

始终坚持廉洁从政。全面落实政府系统党风廉政建设责任制，严格执行廉洁从政各项规定。严格执行国务院“约法三章”，做到政府性楼堂馆所一律不得新建和改扩建，财政供养人员总量只减不增，“三公经费”只减不增。加强效能建设，确保市委、市政府重大决策不折不扣落到实处。加大行政监察，引深政风行风评议工作，坚决查处公职人员吃拿卡要和不作为、乱作为，破坏吕梁发展环境的典型案件。强化审计监督，将审计工作覆盖财政投资建设项目全过程，用审计推动廉洁政府建设。

切实转变工作作风。充分运用党的群众路线教育实践活动成果，大力弘扬焦裕禄精神，聚焦作风建设，狠刹“四风”之害，补足精神之“钙”，大力整治“庸懒散奢”等不良风气；严格执行中央“八项规定”，进一步密切联系群众，切实转变工作作风，改进会风文风，着力解决关系群众切身利益和联系服务群众“最后一公里”问题。进一步加强队伍建设，践行“三严三实”要求，坚定理想信念、筑牢宗旨意识、强化责任意识、提高自觉意识，踏踏实实做好各项工作，努力建设一支政治过硬、思想过硬、作风过硬、能力过硬的干部队伍。

各位代表，空谈误国、实干兴邦，落实才是硬道理。今年政府工作要全面落实市委“打基础、利长远、惠民生”的总体要求，求真务实、关注民生、真抓实干、务求实效。要牢固树立宗旨意识。时刻把群众冷暖安危挂在心上，时刻把促进吕梁经济社会平稳发展和改善民生作为工作的出发点和落脚点。要不断强化责任担当。进一步推行领导干部安全生产、项目建设、扶贫攻坚、城镇化建设、廉政建设“五位一体”包联机制，把“一岗多责”落到实处，真正做到“三明确、三到位”，即：任务明确、要求明确、时限明确；责任到位、措施到位、追究到位，建立严格的责任倒逼、责任倒查、责任追究机制，严肃追查不落实的事、严厉追究不落实的人。要大力弘扬实干精神。以“踏石留印”的劲头和胆魄，转作风、抓落实；以“责无旁贷”的勇气和担当，重民意、惠民生；以“功不在我”的胸怀和理念，促发展、利长远，夙夜在公、勤勉工作，扎扎实实做好每一项工作、踏踏实实办好每一件实事。

各位代表，人民赋予重托，目标催人奋进。让我们在中共吕梁市委的坚强领导下，紧紧依靠全市人民，改革创新、锐意进取，攻坚克难、奋力拼搏，为加快实现稳定脱贫、转型跨越发展、全面建成小康社会的宏伟目标而努力奋斗！
